# Infrared Plasmonic Refractive Index Sensor with Ultra-High Figure of Merit Based on the Optimized All-Metal Grating

**DOI:** 10.1186/s11671-016-1773-2

**Published:** 2017-01-03

**Authors:** Ruifang Li, Dong Wu, Yumin Liu, Li Yu, Zhongyuan Yu, Han Ye

**Affiliations:** 1 State Key Laboratory of Information Photonics and Optical Communications, Beijing University of Posts and Telecommunications, 100876 Beijing, China; 2 School of Science, Beijing University of Posts and Telecommunications, 100876 Beijing, China

**Keywords:** Metamaterials, Optical sensing and sensors, Surface plasmons, Absorption

## Abstract

A perfect ultra-narrow band infrared metamaterial absorber based on the all-metal-grating structure is proposed. The absorber presents a perfect absorption efficiency of over 98% with an ultra-narrow bandwidth of 0.66 nm at normal incidence. This high efficient absorption is contributed to the surface plasmon resonance. Moreover, the surface plasmon resonance-induced strong surface electric field enhancement is favorable for application in biosensing system. When operated as a plasmonic refractive index sensor, the ultra-narrow band absorber has a wavelength sensitivity 2400 nm/RIU and an ultra-high figure of merit 3640, which are much better than those of most reported similar plasmonic sensors. Besides, we also comprehensively investigate the influences of structural parameters on the sensing properties. Due to the simplicity of its geometry structure and its easiness to be fabricated, the proposed high figure of merit and sensitivity sensor indicates a competitive candidate for applications in sensing or detecting fields.

## Background

Recently, the plasmonic metamaterial absorbers based on the surface plasmon resonance (SPR) have attracted increasing attention due to their potential extensive applications in energy harvesting [1–3], hot-electron collection [4–7], thermal emitters [8–11], and biosensors [12–17]. Up to date, various plasmonic metamaterial absorbers have been proposed in different wavebands, which are mainly divided into two main categories: broadband absorbers and narrowband absorbers. As we all know, broadband absorbers are usually applied in solar energy harvesting and thermal emission, while narrowband absorbers are generally used in detectors and sensors.

It is generally known that the sensing performance are usually evaluated by wavelength sensitivity (S) and figure of merit (FOM), which are defined as *S* = *Δλ*/*Δn* and FOM = *S*/FWHM, respectively. *Δλ* is the variation of the resonance wavelength induced by the refractive index change *Δn*. The FWHM is the full width at half maximum of the absorption spectrum. For a plasmon sensor, the key parameter of high FOM means high sensitivity and high wavelength resolution. So far, numerous plasmonic refractive index sensors based on the various nanostructures have been reported, among them, the plasmonic refractive index sensors based on the narrowband absorbers have obtained significant progresses. However, the further applications of the previously published plasmonic sensors are still limited by their relatively complex geometries and low FOM value [18–35]. Zhengqi Liu et al. numerically investigated a sensor based on multispectral ultra-narrowband absorber. This sensor has a wavelength sensitivity exceeding 1000 nm/RIU and FOM of only 5 [25]. Liu et al. demonstrated theoretically a plasmonic sensor based on the plasmonic structure with network-type metasurface on the two-layer dielectric films, which have the FOM of 68.57 [26]. Using Au bowtie nanoantenna arrays with metal-insulator-metal configuration, Lin simulated numerically a sensor with the FOM of 254 [27]. With the efforts made by Luo, the relatively higher FOM of 1054 has been obtained via a metal-metal-dielectric-metal (MMDM) structure [28]. Lu proposed a metal nanobar array with nanoslits backed metal plate structure in theory, which has FOM reaching 25 [29]. Thus, it is desired to propose a simple plasmonic sensor with ultra-high FOM.

In this paper, we demonstrate a simple perfect infrared plasmonic metamaterial absorber based on the all-metal-grating structure [36–39]. Its absorption efficiency is over 98% at normal incidence. Operated as a plasmonic refractive index sensor, the structure has a high wavelength sensitivity 2400 nm/RIU and an ultra-narrow FWHM 0.66 nm. The FOM can reach to 3640, which is far higher than that of the similar published sensors [18–35]. Furthermore, we also investigate the sensing properties of the structure by scanning the structural parameters, such as the length and depth of two nanogrooves. In general, the proposed sensor is not only simple in geometry structure but also exhibits better sensing performance, which means great potential applications in biosensing and detecting areas.

## Methods

**Fig. 1:**
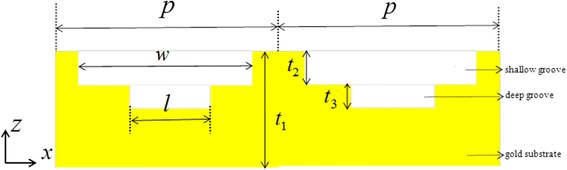
Schematic of the proposed metamaterial structure of a unit cell

Figure 1 illustrates the schematic of the designed all-metal-grating structure. The dimensional parameters are as follows: the periodic length of one unit cell along *x*-axis *p*, gold substrate thickness *t*_1_, the shallow nanogroove width *w*, and height *t*_2_, the deep nanogroove width *l*, and height *t*_3_. The metal material is gold. In the near-infrared, the relative permittivity of gold can be well described by the Drude model *ε*_*m*_ = *ε*_∞_ − *w*_*p*_^2^/[*ω*(*ω* + *iγ*)], where *w*_*p*_ is the plasma frequency and its value is *w*_*p*_ = 2*π* × 2175 THz. *γ* is the damping constant related to the electrons and its value is *γ* = 2*π* × 6.5 THz.

The numerical simulation for the optical performance of the proposed structure is executed by using two-dimension finite-difference time-domain (FDTD) method. We set periodic boundary conditions in the *x* direction, and the incident plane wave is normal to the surface of the proposed structure with the electric field polarized along the *x* direction.

## Results and Discussion

**Fig. 2:**
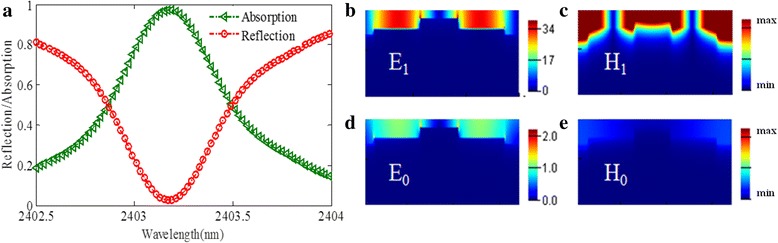
**a** Absorption and reflection spectra of the proposed sensor. Distributions of **b** the electric field *E*_1_ and **c** the magnetic field *H*_1_ at the resonant wavelength. Distributions of **d** the electric field *E*_0_ and **e** the magnetic field *H*_0_ at the nonresonant wavelength. Parameters: *p* = 1200 nm, *w* = 622 nm, *l* = 420 nm, *t*_1_ = 328 nm, *t*_2_ = 28 nm, and *t*_3_ = 18 nm

The transmission light is completely suppressed by the thicker gold substrate (*T* = 0), so the absorption can be defined as *A* = 1 − *R* (reflection). In Fig. 2a, the green and red lines, respectively, present the absorption and reflection spectra. The green line demonstrates that at the resonance wavelength 2403.15 nm, the peak absorption could exceed 98%. The FWHM of the proposed structure is only 0.66 nm, which is far narrower than that of the published narrowband absorbers [18–35]. The structure parameters are listed in the figure caption.

To clearly explain the physical mechanism of ultra-narrow band light absorption of the all-metal-grating structure, we examine the distribution patterns of the electric field E_1_ and magnetic field H_1_ at the resonant wavelength 2403.15 nm, which are depicted in Fig. 2b, c, respectively. Besides, we also calculate and present the snapshots of the electric field distribution E_0_ and the magnetic field distribution H_0_ at the nonresonant wavelength 2423.31 nm for comparison. Note that enhanced field intensity on the surface of the proposed structure can be observed, which is due to the excitation of surface plasmon resonances (SPR). The electric field intensity at the resonant frequency is 40 times larger than that of the incident waves, which is a key point in biosensing applications.

**Fig. 3:**
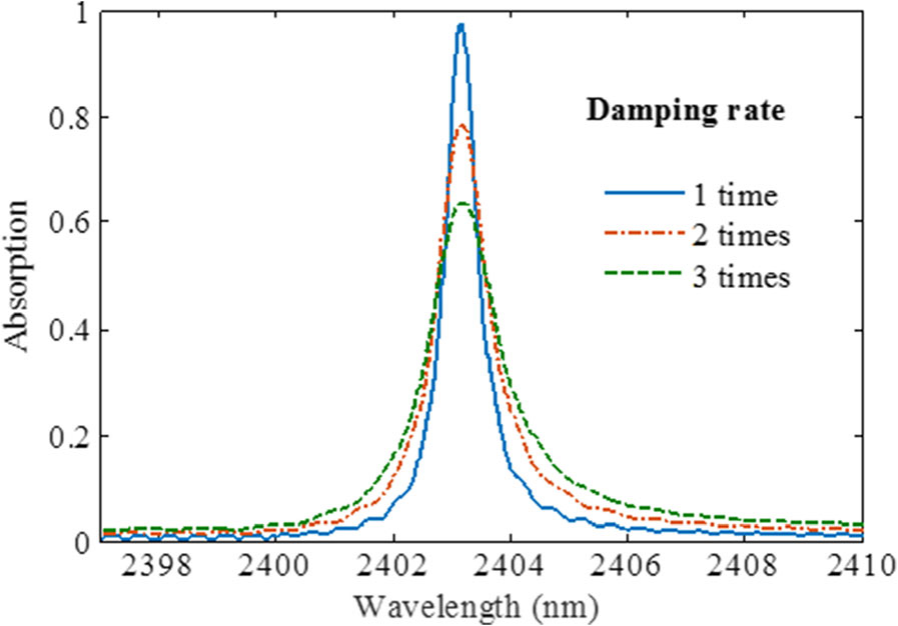
Simulated absorption spectra for damping constant of one, two, and three times that of gold

As shown in Fig. 2b, c, the field distributions above the metal grating structure exhibits a slow attenuation, and only a small proportion of electromagnetic field penetrates into the metal grating, which implies a very small resistive rate. So the total damping rate under critical coupling condition is ultra-small, resulting in ultra-narrow band of the perfect absorber. To prove this, the absorption spectra are compared and analyzed among the structures, which are made of gold with different damping rates *γ*. As shown in Fig. 3, obviously, a decreased and broadened absorbance is observed as the damping rate of gold increases with the same geometrical parameters.

### Properties and Performance

The proposed perfect absorber can be used as a sensor to detect the variation of refractive index of the surrounding dielectric environment, owing to its ultra-narrow bandwidth and ultra-low reflectivity dip. As is well known, the sensing properties are very closely related with the structure. In the following, we simulate numerically about the sensing properties, which include the resonance wavelength, the reflectivity dip, FWHM, and FOM by scanning the structural parameters such as the shallow nanogroove width *w*, height *t*_2_, the deep nanogroove width *l*, and height *t*_3_. In simulation, we assume that all variables other than the one under consideration are kept constant, and are listed in corroding the figure caption. In practical applications, it is necessary to detect the relative intensity change *dI*/*dn* at a fixed wavelength due to the refractive index change [18]. The corresponding figure of merit can be defined as FOM* = max |(*dI*/*dn*)/*I*| [20].

**Fig. 4:**
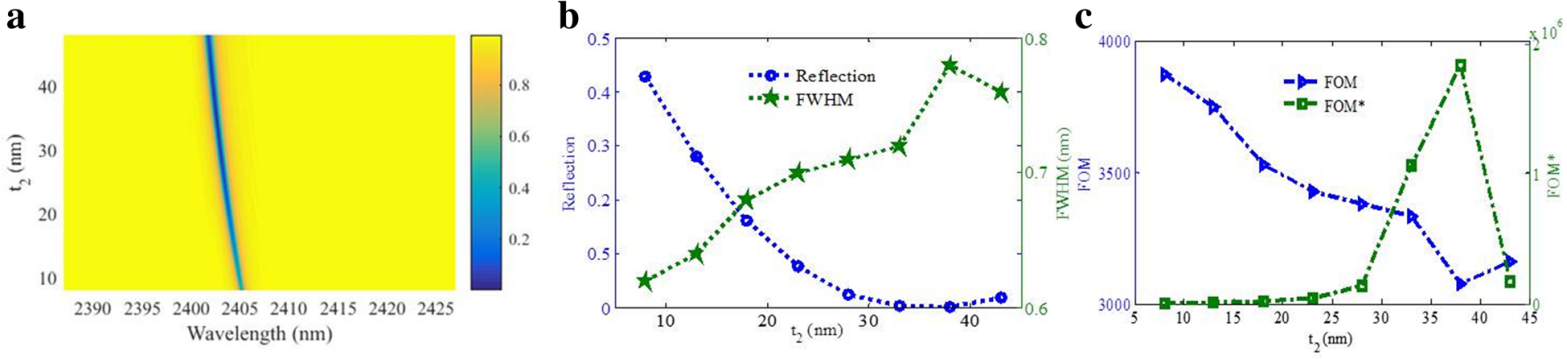
**a** Reflection spectra as a function of *t*_2_. **b** Reflectivity of the resonance dip and its FWHM as functions of *t*_2_. **c** FOM and FOM* as functions of *t*_2_. Other parameters: *p* = 1200 nm, *w* = 622 nm, *l* = 420 nm, *t*_3_ = 18 nm, and *t*_1_ = 300 nm + *t*_2_

Figure 4a, b shows the reflection properties as functions of the shallow nanogroove height *t*_2_. As *t*_2_ changes from 8 to 48 nm, the resonance peak will blueshift. At the same time, the FWHM will become wider. When the shallow nanogroove height *t*_2_ is interval of 30–40 nm, there is a minimum reflectivity dip. Based on the definition of FOM, the FOM will decrease as shown in Fig. 4c owing to the wider FWHM. The FOM* as function of *t*_2_ is shown in Fig. 4c. The maximum value of FOM* can reach 1.83 × 10^6^ with the *t*_2_ of around 38 nm, which gives us a guide in designing of high-quality plasmonic refractive index sensor.

The variational trend of the sensing properties with the increasing of *t*_3_ is similar to that of *t*_2_ except that the resonance wavelength exhibits redshift. And it is easy to observe that the FWHM is more influenced by *t*_3_. The FOM decreases obviously and has a maximum value of 6315.8, which is much higher than that of the previously reported plasmonic refractive index sensor [18–35]. However, when the maximum FOM is obtained, the value of the reflectivity dip approaches 0.3, which is too high to be easily detected. Thus, we need to find a compromise method to obtain a higher FOM and lower reflectivity dip simultaneously.

Figure 6 presents the effects of the shallow nanogroove width *w* on the sensing performance. As shown in Fig. 6a, the resonance wavelength keeps nearly unchanged with changing of *w*. Figure 6b shows that when the *w* is 620 nm, the minimum values of the reflectivity dip and the FWHM can be simultaneously obtained, which is favorable to obtain the high-quality sensors. At the same time, the maximum FOM can reach to 3640 when the *w* is 620 nm.

As shown in Fig. 7a, as the deep nanogroove width *l* increases from 340 to 500 nm; the resonance wavelength exhibits no-shift just as in Fig. 6a. According to Fig. 7b, the reflectivity dip will decrease, while the FWHM will increase as the increasing of *l*. The FWHM changes slightly and the reflectivity dip sustains an ultra-low value under 0.06, which are important to practical application due to its robustness. The maximum FOM exceeding 3871 can be obtained when *l* is 360 nm.

The resonance wavelength can be explained by the equivalent LC circuit model of the metamaterial structure [40–45]. The resonance wavelength can be given by $\lambda =2\pi c\sqrt{\mathrm{LC}}$. The gap capacitance *C* can be represented as *C* = *ε*_0_ *S*/*d*, where *ε*_0_ is the dielectric permittivity of the surrounding environment and *S* is the effective area of the capacitance. *d* is the effective width of the nanogrooves. The inductance of the gold substrate is expressed as *L*, which is inversely proportional to the volume of metallic bars. In the LC circuit model, we can qualitatively predict the effect of the structural parameters on the resonance wavelength.

As increasing of *t*_2_, the effective area of the capacitance *C* will increase. However, the effective inductance *L* will decrease linearly with increasing of *t*_2_, owing to the increase in volume of metallic bars, which will cause decreasing of resonance wavelength. In the designed structure, the shallow nanogroove *w* is much wider, which will result in the extremely slight effect of *t*_2_ on the capacitance *C*. In other words, the increasing volume of metallic bars that induced variation of *L* perhaps have more influences on the position of the resonance frequency than that of the decreasing of effective area that induced the changing of *C*. Thus, the resonance wavelength will blueshift slightly with increasing *t*_2_, which matches the simulated result in Fig. 4a quite well.

**Fig. 5:**
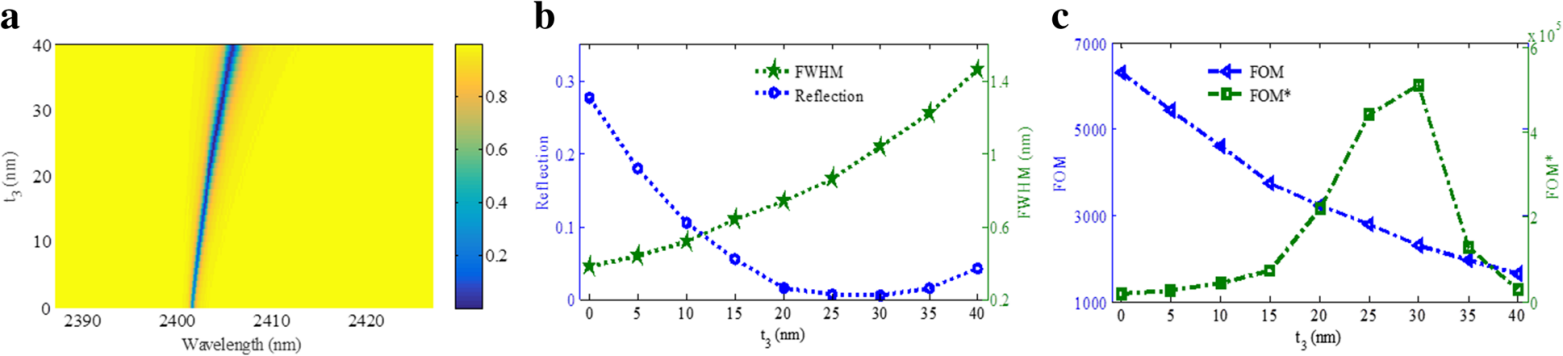
**a** Reflection spectra as a function of *t*_3_. **b** Reflectivity of the resonance dip and its FWHM as functions of *t*_3_. **c** FOM and FOM* as functions of *t*_3_. Other parameters: *p* = 1200 nm, *w* = 622 nm, *l* = 420 nm, *t*_1_ = 328 nm, and *t*_2_ = 28 nm

With the increasing of *t*_3_, the *C* will be enhanced, which will increase the resonance wavelength of the SPR mode. At the same time, a larger *t*_3_ will lead to a smaller volume of metallic bars, which will result in the increase of *L*. Considering the influences of the two parameters, the resonance wavelength will redshift slightly with increasing *t*_3_. The qualitatively predicted results of the *LC* model and the simulated results (shown in Fig. 5a) are coincident.

**Fig. 6:**
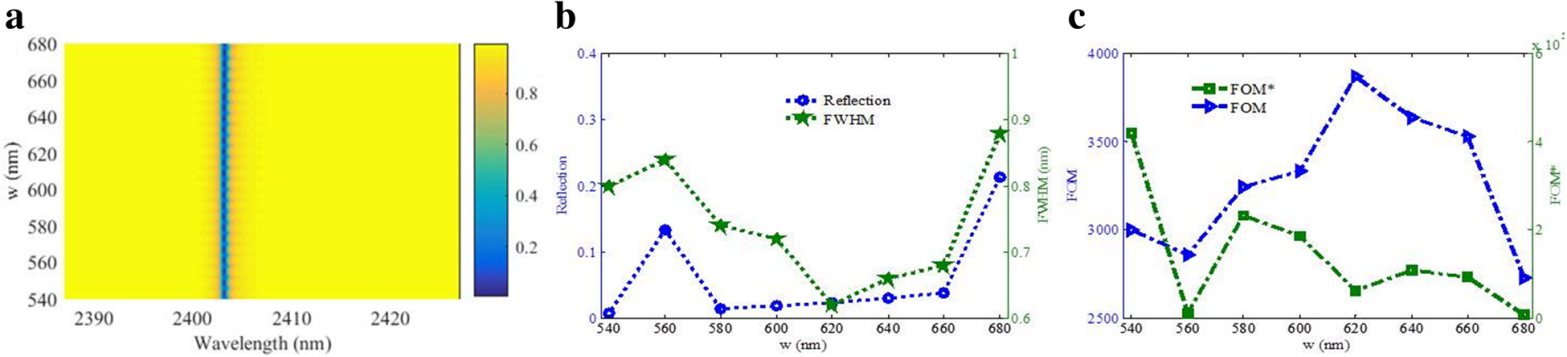
**a** Reflection spectra as a function of *w*. **b** Reflectivity of the resonance dip and its FWHM as functions of *w*. **c** FOM and FOM* as functions of *w*. Other parameters: *p* = 1200 nm, *l* = 420 nm, *t*_1_ = 328 nm, *t*_2_ = 28 nm, and *t*_3_ = 18 nm

**Fig. 7:**
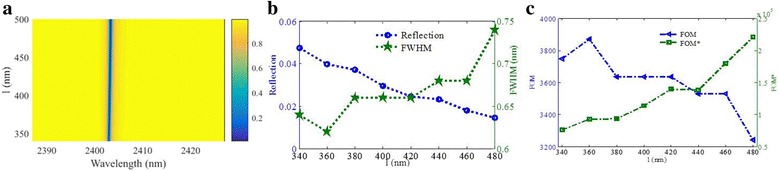
**a** Reflection spectra as a function of *l*. **b** Reflectivity of the resonance dip and its FWHM as functions of *l*. **c** FOM and FOM* as functions of *l*. Other parameters: *p* = 1200 nm, *w* = 622 nm, *l* = 420 nm, *t*_1_ = 328 nm, *t*_2_ = 28 nm, and *t*_3_ = 18 nm

The volume of the metal will decrease as in the increasing of *w*, which will cause larger *L*. Thus, the resonance wavelength will redshift. However, *C* will decrease linearly with the increasing of *w*, which will induce the blueshift of the resonance wavelength. The effects of the two parameters on the resonance wavelength can be counteracted. Thus, the position of the resonance keeps nearly unchanged, which is consistent with the simulation results shown in Fig. 6a. Similarly, as in the increasing of *l*, the resonance wavelength will change little shown in Fig. 7b. In practical application, this robustness is beneficial to structure design.

**Fig. 8:**
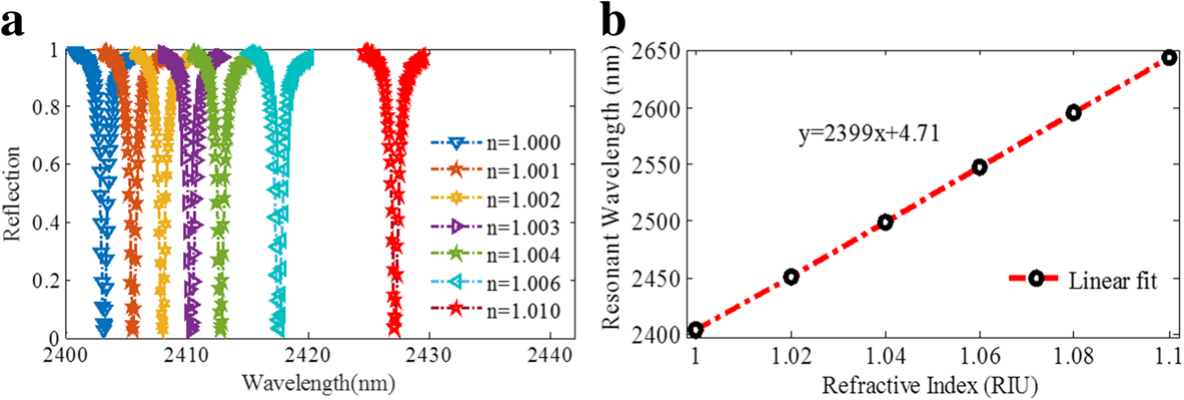
**a** Reflection spectra of the sensor with various refractive indexes of the environment. **b** Resonant wavelength shifts against the surrounding refractive index. Parameters: *P* = 1200 nm, *w* = 622 nm, *l* = 420 nm, *t*_1_ = 328 nm, *t*_2_ = 28 nm, and *t*_3_ = 28 nm

Based on the discussion above, in the following, we give a detailed analysis of the metamaterial structure as a refractive index sensor with various structure parameters. The structure parameters are shown in the caption of Fig. 8. We investigate the sensing properties of the absorber with various refractive index of the environment. According to Fig. 8a, the resonant wavelength will redshift as the surrounding refractive index varies from 1.000 to 1.010. At the same time, the reflectivity dip can sustain an ultra-low value. As shown in Fig. 8b, the proposed plasmonic refractive index sensor has a high linearity over a wide range of wavelength. For example, as the refractive index ranges from 1.00 (vacuum) to 1.06 with intervals of 0.02, the resonant wavelength is obtained at 2403.15, 2451.18, 2499.19, and 2547.18 nm, respectively. The slope of the fitting line represents the sensitivity (S) reaching 2400 nm/RIU. Meanwhile, the FWHM can be narrower than 0.66 nm. By the definition of FOM, the FOM of the proposed plasmonic sensor can reach up to 3640, which is much larger than that of the previously published similar sensors [18–35].

## Conclusions

In conclusion, we propose and demonstrate a perfect near-infrared plasmonic metamaterial absorber with a simple all-metal-grating structure by using a two-dimensional FDTD method. The structure shows that an ultra-narrow absorption bandwidth of 0.66 nm and the absorption efficiency over 98% are obtained. The high absorption with ultra-narrow bandwidth is mainly ascribed to the excitation of surface plasmon resonances and the ultra-small total damping rate under critical coupling condition. And the structure can obtain the giant surface electric field enhancement. Owing to its ultra-narrow bandwidth and ultra-low reflectivity dip, the absorber can be used as a sensor. We numerically investigate the effects of dimensional parameters on the properties of the sensor in detail. As a plasmonic refractive index sensor, the structure can present excellent performance with S of 2400 nm/RIU and FOM of 3640 in a wider spectral range. For its above-mentioned merits, especially the ultra-high S and ultra-high FOM, the plasmonic refractive index sensor with simple structure could provide a promising approach for gas detection, biosensors, and medical diagnostics.

## Abbreviations

FDTD: Finite-difference time-domain
FOM: Figure of merit
FWHM: Full width at half maximum
S: Sensitivity
SPR: Surface plasmon resonance
